# Potential role of CXCL9 induced by endothelial cells/CD133+ liver cancer cells co-culture system in tumor transendothelial migration

**DOI:** 10.18632/genesandcancer.116

**Published:** 2016-07

**Authors:** Qiang Ding, Yujia Xia, Shuping Ding, Panpan Lu, Liang Sun, Yuhui Fan, Xin Li, Ying Wang, De-an Tian, Mei Liu

**Affiliations:** ^1^ Institute of Liver Diseases, Department of Gastroenterology, Tongji Hospital, Tongji Medical College, Huazhong University of Science and Technology, Wuhan, Hubei Province, China; ^2^ Department of Gastroenterology, the Central Hospital of Wuhan, Tongji Medical College, Huazhong University of Science and Technology, Wuhan, Hubei Province, China

**Keywords:** CD133+, liver cancer cells, HUVEC, co-culture system, CXCL9

## Abstract

Transendothelial migration is a pivotal step before the dissemination of tumor cells into the blood circulation. Related researches about the crosstalk between tumor cells and endothelial cells could contribute to understanding the mechanism of transendothelial migration. Cumulative studies showed that CD133 was an important marker for cancer stem cells. In our research, a co-culture system was developed to study the interaction between CD133+ liver cancer cells and human umbilical vein endothelial cells. The results showed that the direct co-cultured supernatants promoted the migration and invasion of CD133+ liver cancer cells. It was further investigated that the expression level of chemokine CXCL9 was significantly elevated in the culture supernatants of direct co-culture system by activating the NF-kB, rather than in the indirect co-culture system or mono-culture system. High expression of CXCL9 in the direct co-cultured supernatants played a significant role in enhancing the migration and invasion of CD133+ liver cancer cells. Collectively, these findings suggest that chemokine CXCL9 may function as a potential target during the process of transendothelial migration.

## INTRODUCTION

Hepatocellular carcinoma (HCC) is the fifth most common cancer and the second leading cause of cancer- associated deaths, which is over 600,000 around the world [1]. The poor prognosis with overall survival rates of 3-5% is mainly due to the distant metastasis [2]. However, the development of a metastatic trait is rather complex, but the process generally consists of the following phases: primary cancer cell migration and invasion, transendothelial migration (TEM) into the circulation, extravasation, and distant seeding [3]. No matter how tumor cells enter or leave blood vessels, the interaction between tumor cells and endothelial cells is of great importance to TEM.

Liver cancer stem cells (CSCs) are postulated as a small subset of undifferentiated cells in liver cancer. They mirror the characteristics of embryonic or pluripotent stem cells, and they are responsible for liver cancer relapse and chemoresistance [4, 5]. CD133 cell surface glycoprotein has been reported as an important marker for liver cancer stem cells and is used for the identification and isolation of CSCs from liver cancer tissues as well as cell lines [6, 7].

In this study, Our findings demonstrate that the supernatants in the direct co-culture system significantly promotes the migration and invasion abilities of the CD133+ liver cancer cells than that of both in the indirect co-culture system and the mono-culture. Chemokine CXCL9 is a small secreted protein and belongs to the member of C-X-C subfamily [8, 9]. It is further determined that CXCL9 expression in the supernatant of the direct co- culture system is upregulated via the activation of NF-kB. Furthermore, CXCL9 was identified to play a key role in the migration and invasion of CD133+ liver cancer cells. Above all, the results suggest that high CXCL9 release may play a key role in tumor TEM due to enhancing the migration and invasion abilities of the CD133+ liver cancer cells.

## RESULTS

### The supernatants in the direct co-culture system of HUVEC and CD133+ liver cancer cells promoted the metastatic properties of CD133+ liver cancer cells

**Figure 1:**
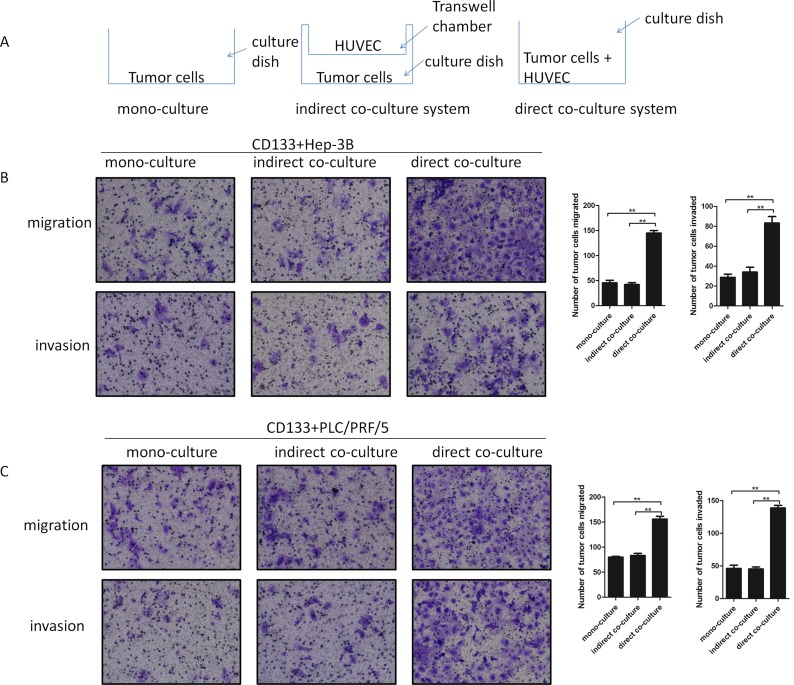
The migration and invasion abilities of CD133+ liver cancer cells (including CD133+ Hep-3B and CD133+ PLC/PRF/5) were enhanced when added to the direct co-cultured supernatants (A) The cartoon diagram was used to delineate the co-culture system simply. (B) and (C): Representative images of Transwell assays to assess cell migration and invasion abilities. (Data are represented as the mean ± SEM, **P < 0.01).

In order to investigate the interaction between HUVEC and the CD133+ liver cancer cells (including the CD133+ PLC/PRF/5 and Hep-3B cells), both the indirect and direct co-culture systems were used. The supernatants from co-culture systems were used as the chemotaxis factors for transwell assays. It was found that the supernatants of the direct co-culture groups facilitated the migration and invasion of CD133+ liver cancer cells more than that of the indirect co-culture group and mono-culture group (Fig 1B, 1C). Moreover, the transwell assays results showed no statistical difference between the indirect co- culture and mono-culture group (Fig 1B, 1C). Therefore, the results above suggested that when the CD133+ liver cancer cells contacted with HUVEC, some cytokines were produced that could promote the migration and invasion of CD133+ liver cancer cells.

### The proliferation of CD133+ liver cancer cells increased when directly co-cultured with HUVEC

**Figure 2:**
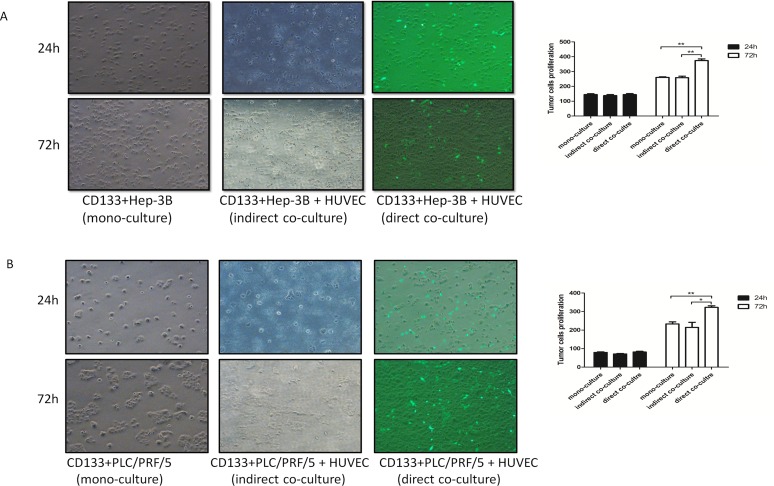
The proliferation capacities of CD133+ liver cancer cells (including CD133+ Hep-3B and CD133+ PLC/ PRF/5) were enhanced when directly co-cultured with human umbilical vein endothelial cells (HUVEC) (A) and (B): Representative images depicted the proliferation of CD133+ liver cancer cells in co-culture system using fluorescence microscopy. The numbers of CD133+ liver cancer cells (not expressing eGFP) were quantified. (Data are represented as the mean ± SEM, * P < 0.05, ** P < 0.01).

The HUVEC cell line stably expressed eGFP through lentivirus-eGFP infection to distinguish from the CD133+ liver cancer cells under fluorescence microscopy. As the enhanced migration and invasion capacities are always accompanied with increasing proliferation ability, it was found that the proliferative effect of the CD133+ liver cancer cells increased in direct co-cultured group more than that in indirect co-culture and mono-culture groups (Fig 2A, 2B). Moreover, the proliferation ability between the in-direct and mono-culture groups showed no obvious difference (Fig 2A, 2B). Taken together, it suggested that the direct interaction was of great importance for the proliferative effect of CD133+ liver cancer cells.

### CXCL9 expression was upregulated in the supernatant of direct co-culture system via the activation of NF-kB and promoted tumor migration and invasion

**Figure 3:**
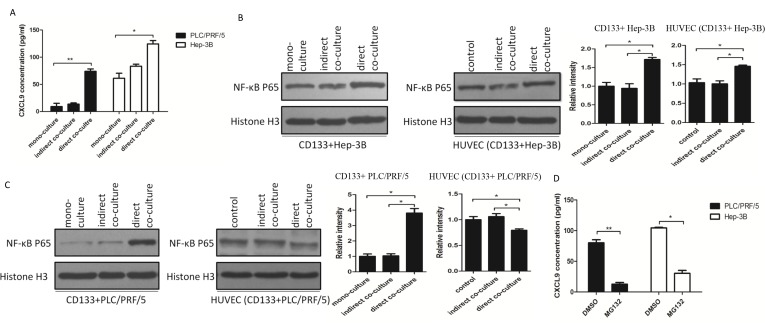
CXCL9 expression was upregulated in direct co-cultured supernatants through the activation of NF-kB (A) ELISA assays were performed to detect the CXCL9 expression. (B) and (C): The western blot results showed that NF-kB signaling pathway was activated in CD133+ liver cancer cells of the direct co-culture group. NF-kB was upregulated in HUVEC (CD133+ Hep-3B), but downregulated in HUVEC (CD133+ PLC/PRF/5). (D) The ELISA results showed that the CXCL9 expression reduced significantly in the supernatants of direct co-culture group treated with NF-kB inhibitor MG132. (Data are represented as the mean ± SEM, * P < 0.05, ** P < 0.01).

**Figure 4:**
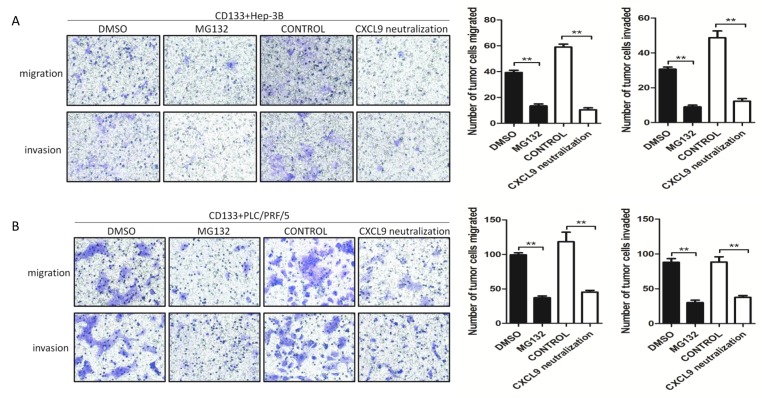
The migration and invasion abilities of CD133+ liver cancer cells attracted by the direct co-cultured supernatants decreased when the direct co-culture groups were pretreated with MG132 or the supernatants were pretreated with CXCL9 neutralizing antibody (A) and (B): Representative images of transwell assays to show the migration and invasion abilities of CD133 liver cancer cells. The numbers of invasive and migratory cells were quantified. (Data are represented as the mean ± SEM, * P < 0.05, ** P < 0.01).

The Enzyme-Linked Immunosorbent Assay (ELISA) was performed to identify the CXCL9 expression level. The results revealed that chemokine CXCL9 was more pronouncedly secreted in the supernatant of direct co-culture group than that of indirect co-culture and mono- culture groups (Fig 3A). NF-kB belongs to the Rel family of transcription factors, which could regulate a large number of inflammatory genes [10]. To determine whether NF-kB upregulated CXCL9 release in the direct co-culture system, CD133+ liver cancer cells were isolated from the mixed co-cultured cell line by fluorescence-activated cell sorting and western blot was used to detect the nuclear NF-kB p65 protein expression. The results showed that nuclear NF-kB p65 protein was highly upregulated in the CD133+ liver cancer cells from the direct co-culture groups. Also, nuclear NF-kB p65 protein was upregulated in HUVEC isolated from the HUVEC/CD133+Hep-3B direct co-culture system. However, nuclear NF-kB p65 protein decreased in HUVEC isolated from the HUVEC/ CD133+PLC/PRF/5 direct co-culture system (Fig 3B, 3C). When the direct co-culture groups were treated with the NF-kB inhibitor MG-132, the ELISA data exhibited remarkable reduction on CXCL9 expression (Fig 3D). The results above suggested that NF-kB was closely related with the CXCL9 expression in direct co-culture system. Regardless of whether HUVEC enhanced or reduced CXCL9 secretion when directly co-cultured with CD133+Hep-3B or CD133+PLC/PRF/5 respectively, CXCL9 secretion in the supernatants of the direct co- culture groups was still upregulated, which suggested that CXCL9 was mainly secreted by direct co-cultured CD133+ liver cancer cells. And it was further found that the chemotaxis abilities of supernatants from the direct co-culture groups treated with MG132 decreased significantly (Fig 4). Moreover, the chemotaxis capacities of direct co- cultured supernatants decreased obviously when pretreated with CXCL9 neutralizing antibody (Fig 4). These findings suggested that CXCL9 could be significantly upregulated through the activation of NF-kB signaling pathway and played a key role in migration and invasion of CD133+ liver cancer cells.

## DISCUSSION

Hepatocellular carcinoma is often accompanied by high cancer-related mortality [1]. Distant metastasis is a great challenge that we face when fighting cancer, a life-threatening disease. During the process of cancer metastasis, the transendothelial migration of tumor cells is a critical step [3]. Thus, related research about the crosstalk between the endothelial cells and tumor cells becomes very significant. Liver cancer stem cells are a crucial population in liver cancer, which have the capacity to self-renew and differentiate into other cells to maintain tumor homeostasis [11]. Although there are many distinct cell surface proteins that are evaluated as CSCs markers, CD133 plays a regulatory role in liver cancer [12]. There exist studies that focus on the potential biological behavior of CD133+ liver cancer cells [13–16]. In this research, we found that the direct co-cultured supernatants were able to enhance the migration and invasion of CD133+ liver cancer cells. Previously, our team mainly focuses on the role of CXCL9 in liver diseases including hepatitis and liver cancer and found that CXCL9 is involved in them. To further investigate the underlying mechanism, chemokine CXCL9 was been tested by ELISA. The results showed that chemokine CXCL9 was highly upregulated in direct co-culture system. To determine whether NF-kB was implicated in the CXCL9 overexpression in the direct co- culture system, the nuclear NF-kB p65 was detected by western blot and confirmed to participate in this process. When the direct co-culture systems were treated with NF- kB inhibitor MG132, the chemokine CXCL9 secretion in the supernatants decreased remarkably. In addition, when the direct co-cultured supernatants were added with the neutralizing antibody of CXCL9, the enhanced migration and invasion abilities of CD133+ liver cancer cells were abolished. All the data indicated that the supernatants in the direct co-culture system promoted the metastasis of CD133+ liver cancer cells via the upregulated CXCL9 expression through the activation of NF-kB.

In summary, our findings would enrich the knowledge about the interaction between the endothelial cells and tumor cells, especially with the CD133+ liver cancer cells. The results suggest that high expression of CXCL9 will be induced by direct contact of the endothelial cells and tumor cells. Moreover, CXCL9 can promote the migration and invasion of tumor cells. Therefore, it may be speculated that CXCL9 plays a potential role in tumor TEM. Perhaps it would call more attention to the cancer research about the co-culture system between tumor cells and the other cells, such as with the immune cells, the cells of distant target organs, and so on. Taken together, this work might provide a therapeutic target against HCC, particularly for its metastasis.

## MATERIALS AND METHODS

### Cell culture and fluorescence-activated cell sorting

PLC/PRF/5, Hep-3B and HUVEC cell lines were purchased from the American Type Culture Collection and are frozen aliquots after the two weeks of cell expansions. Each aliquot was used for less than 10 personal passages. CD133+ PLC/PRF/5 and Hep-3B cells (hereinafter called CD133+ liver cancer cells) were isolated from PLC/ PRF/5 and Hep-3B liver cancer cell lines by fluorescence- activated cell sorting (FACS) (BD Company, New Jersey, USA) using CD133 antibody (CD133/2(293C3)-APC, 1 test for 107 cells) (Catalog Number: 130-090-854, Miltenyi Biotec, Bergisch Gladbach, Germany). The CD133+ liver cancer cells and human umbilical vein endothelial cells (HUVEC) were cultured in Dulbecco's modified Eagle's medium (DMEM) containing 10% fetal calf serum (Invitrogen Gibco, Carlsbad, CA, US) and incubated at 5% CO2 and 37°C. The HUVEC stably expressed enhanced green fluorescent protein (eGFP). The cells in the direct co-culture system were isolated using FACS-sorted eGFP-positive HUVEC.

### Co-culture systems

CD133+ liver cancer cells and human umbilical vein endothelial cells (HUVEC) were mixed and grown together to establish the direct co-culture system. The two cell lines were seeded and separated by 0.4 μm transwell chamber to establish the indirect co-culture system (Schematic representation in Figure 1A). It is observed lasting for 72 h under serum-free culture systems.

### Western blot

Western blot assays were performed as previous described [17]. NF-kB P65 antibody (1:1000) (Catalog Number: H00005970-M01, Abnova Company, Taiwan, China) was used.

### Enzyme-linked immunosorbent assay (ELISA)

The supernatants from co-culture system were collected and assayed for chemokine CXCL9 protein after 72 h serum-free co-culture systems according to the instruction of the manufacturer (Peprotech Company, NJ, US).

### Cell migration and invasion assay

Matrigel-coated invasion inserts (BD Biosciences, NJ, US) with 8 μm-pored transwell plates (Corning Costar, MD, US) were used for the invasion assays. 600 μl supernatant of different groups in the co-culture system were added to the lower chamber. The CD133+ liver cancer cells (3×104 cells for one chamber) on the upper chamber were removed with a cotton swab, and the CD133+ liver cancer cells on the lower chamber were fixed and stained. Three random fields were chosen to be photographed and counted.
